# Simulation and Validation of Droplet Generation Process for Revealing Three Design Constraints in Electrohydrodynamic Jet Printing

**DOI:** 10.3390/mi10020094

**Published:** 2019-01-29

**Authors:** Yanqiao Pan, Liangcai Zeng

**Affiliations:** 1 Key Laboratory of Metallurgical Equipment and Control Technology of Ministry of Education, Wuhan University of Science and Technology, Wuhan 430081, China; zengliangcai@wust.edu.cn; 2 Hubei Key Laboratory of Mechanical Transmission and Manufacturing Engineering, Wuhan University of Science and Technology, Wuhan 430081, China

**Keywords:** electrohydrodynamic jet printing, droplet generation process, numerical simulation, experimental validation, design constraint, computational fluid dynamics, multi-physics

## Abstract

Droplet generation process can directly affect process regulation and output performance of electrohydrodynamic jet (E-jet) printing in fabricating micro-to-nano scale functional structures. This paper proposes a numerical simulation model for whole process of droplet generation of E-jet printing based on the Taylor-Melcher leaky-dielectric model. The whole process of droplet generation is successfully simulated in one whole cycle, including Taylor cone generation, jet onset, jet break, and jet retraction. The feasibility and accuracy of the numerical simulation model is validated by a 30G stainless nozzle with inner diameter ~160 μm by E-jet printing experiments. Comparing numerical simulations and experimental results, period, velocity magnitude, four steps in an injection cycle, and shape of jet in each step are in good agreement. Further simulations are performed to reveal three design constraints against applied voltage, flow rate, and nozzle diameter, respectively. The established cone-jet numerical simulation model paves the way to investigate influences of process parameters and guide design of printheads for E-jet printing system with high performance in the future.

## 1. Introduction

Recently, electrohydrodynamic jet (E-jet) printing [1,2,3,4] has been considered as a candidate to substitute traditional inkjet printing [5,6] for fabricating micro scale functional structures [7,8,9] that can be widely used in display [10], radio frequency identification (RFID) [11], and flexible sensors [12,13]. There are three arguments: (1) E-jet printing has ability to achieve ultrahigh resolution (~50 nm) [14]; (2) E-jet printing is compatible with viscosity from 1 mPa·s to ~10,000 mPa·s [15,16]; (3) E-jet printing pulls a jet from nozzle orifice by electrical field force, and the jet has much a smaller size than the nozzle diameter [17,18]. Moreover, e-jet printing is a typical technique that is affected by coupled multi-physics [19]. The formation, flying, and deposition of the jet have significant impacts on printing resolution and positioning accuracy [20]. The processes are influenced by interactions of electric field force, gravity, capillary force, viscous force, air resistance, and surface tension. Droplet generation in electrohydrodynamic jet printing can be affected by multiple factors, e.g., nozzle structure, process parameters, and solution properties [21,22,23]. Therefore, it is challenging and meaningful to investigate drop generation behavior in E-jet printing, which provides theoretical guidance for regulation of process parameters and design of printheads with high performance.

At the beginning, some researchers studied drop generation behavior mainly through experiments. In 1917, Zeleny [24] found a special phenomenon through experiments when a suitable electric field is applied between a hanging droplet and a substrate, the shape of the hanging droplet will change gradually from semi-sphere to cone due to electro-rheological effect, and finally a fine jet will burst from the tip of the cone. To explain this unique phenomenon, scholars also made some efforts on theoretical modelling. In 1964, Taylor [25] first analyzed onset conditions of cone-jets by mathematical modelling, and then proposed [26] the dielectric leakage model with Melcher in 1969. In 1997, Ganan-Calvo et al. [27] established a formula against jet shape, charge distribution, current, and flow rate in the cone-jet mode. Barrero et al. [28] found that conductivity determines electric field shear stress on the surface of the Taylor cone in 1999. Through theoretical modelling, it is possible to obtain some affecting laws when some process conditions are simplified. However, it is quite difficult to obtain a full analytical solution or semi-analytical solution to reveal mechanisms of behaviors in drop generation process.

With the development of numerical methods, such as finite element method (FEM) and finite volume method (FVM), scholars started to write calculation programs or purchase commercial software to simulate and analyze the formation of cone jets. In 2003, Yan et al. [29] established a simulation model under two-dimensional axisymmetric conditions by writing a calculation program, and obtained a cone jet morphology. In 2006, Orest et al. [30] also successfully simulated the shape of a cone jet using Ansys CFX (4.4, Ansys, Canonsburg, PA, USA) and the corresponding user defined function (UDF). They found vortex distribution law in the flow field, but the simulation model was too simple and there was no jet break in the simulation. In 2013, Najjaran [31] and Kim [32] simulated the occurrence of cone jets with Ansys Fluent (12.1, Ansys, Canonsburg, PA, USA) and corresponding UDF, but also did not consider the jet break process. In 2012, Wu et al. [33] simulated the process of cone jets appearing and their fracture and studied the influence of a few nozzle structure parameters on jetting state. At present, there are only few publications on simulation of jet break and retraction and affecting laws about process parameters on printing results. Therefore, it is important to further study the whole droplet generation process and reveal the mechanisms inside in E-jet printing.

In this paper, a numerical simulation model for E-jet printing is proposed based on the Taylor-Melcher leaky-dielectric model and the whole process of droplet generation is successfully simulated in one whole cycle, including Taylor cone generation, jet starting, jet break, and jet retraction using FLOW-3D (9.3.2, Flow Science, Santa Fe, NM, USA). Multiple experiments are also performed to validate the feasibility and accuracy of the numerical simulation model based on a 30G stainless nozzle. Further simulations are performed to reveal three design constraints against applied voltage, flow rate, and nozzle diameter, respectively. This study paves the way to investigate impacts of more process parameters and the guide design of printheads for E-jet printing systems with high performance in the future.

## 2. Numerical Simulation of a Whole Droplet Generation Process

### 2.1. Physical Model

**Figure 1:**
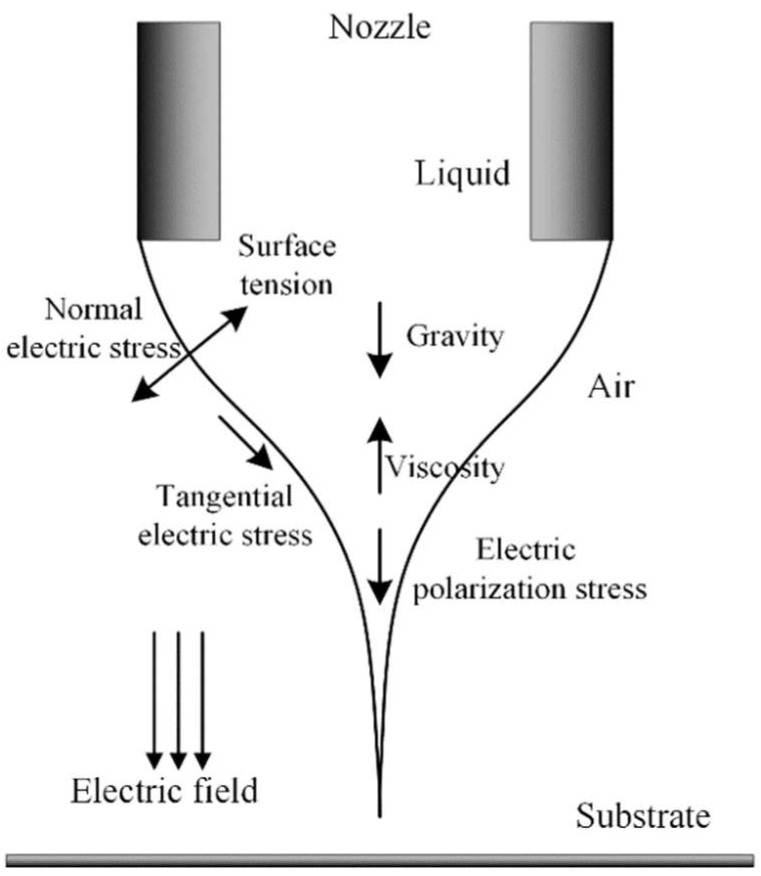
Distribution of active forces on the liquid cone-jet.

E-jet printing is a typical technique that is affected by multiple coupled physical forces, such as hydrodynamic pressure and electrostatic force. Whole process can be achieved when the governing equations for fluid flow and electric field are solved. Our computational fluid dynamics (CFD) calculations for E-jet printing are based on the leaky dielectric model proposed by Taylor and Melcher [26]. In the model, liquid is regarded to be dielectric and free charges can move towards the liquid-gas interface. Shear forces on these interfacial charges drive growth of cone and jet formation. Figure 1 shows the distribution of active forces within liquid cone-jets based on the leaky dielectric model. At the interface, normal electric stress is balanced by surface tension, while viscous flow counterbalances tangential components of electric field stress.

Assuming liquid to be dielectric and incompressible (constant density ρ and constant viscosity η), its motion in an electric field can be described by the continuity and momentum conservation equations, like Equations (1) and (2):

(1) $$\nabla \cdot \vec{v}=0$$

(2) $$\rho \frac{d\vec{v}}{dt}=-\nabla P+\eta \nabla^{2}v+\vec{f_{e}}+\rho \vec{g}$$

where P is pressure, t is time, $\vec{v}$ is fluid velocity, $\vec{f_{e}}$ is electromechanical force that can be assumed as Equation (3):

(3) $${\vec{f}}_{e}=q_{e}\vec{E}$$

where $q_{e}$ is charge density and $\vec{E}$ is electric field. The charge density distribution is determined by Gauss’s law as Equation (4):

(4) $$\varepsilon \nabla \cdot \vec{E}=q$$

where ε is the relative dielectric constant of liquid. Using E=−∇ϕ, the electric body force $\vec{f_{e}}$ can be expressed as Equation (5), where ϕ is the electric potential:

(5) $${\vec{f}}_{e}=-\varepsilon \nabla^{2}\phi \cdot \vec{E}$$

The charge conservation is represented as Equation (6):

(6) $$\frac{\partial q}{\partial t}+\nabla \cdot \vec{J}=0$$

where $\vec{J}$ is current density, which is expressed as Equation (7):

(7) $$\vec{J}=q\vec{v}+\sigma \vec{E}$$

where σ is electrical conductivity of the liquid.

In simulation, tracking of the moving gas-liquid interface is achieved using Volume of Fluid (VOF) method. The basic idea shown in Equation (8) is to introduce a fraction F, which represents a portion of a cell’s liquid, and then makes a judgement by comparing with 0 and 1.

(8) $$F\left(x,y,z,t\right)=\left\{ \begin{array}{l} 0\;(\mathrm{outside}\;\mathrm{the}\;\mathrm{liquid})\\ 1\;(\mathrm{inside}\;\mathrm{the}\;\mathrm{liquid})\\ >0,<1\;(\mathrm{on}\;\mathrm{the}\;\mathrm{interface}) \end{array}\right.$$

To represent the dynamic process of the interface, F fulfils the basic kinematic equation as Equation (9):

(9) $$\frac{dF}{dt}+\vec{v}\cdot \nabla F=0$$

where $\vec{v}$ is velocity of liquid.

### 2.2. Numerical Simulation Model

**Figure 2:**
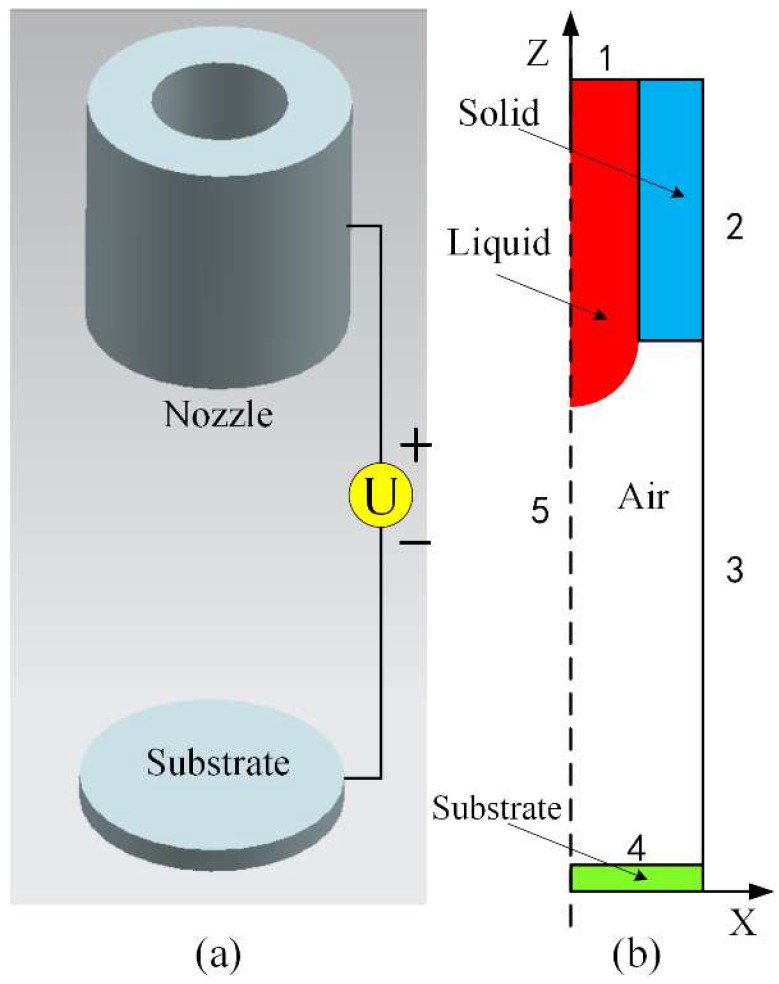
(**a**) Schematic diagram of nozzle-substrate structure, (**b**) schematic diagram of the three-phase calculation model in simulation.

In this part, a numerical simulation model is proposed with details, as shown in Figure 2. Figure 2a shows a schematic diagram of the nozzle-substrate structure, which is used in the numerical simulation model. A 30G stainless nozzle is utilized in the model and the inner and outer diameters of the nozzle are 0.16 mm and 0.31 mm, respectively. The substrate is a single polished Silicon wafer and its dielectric constant is 11.9. The contact angle of the substrate to Ethylene glycol is 80°. The distance of the nozzle to Si-substrate is set to 0.65 mm. The thickness of the substrate is set to 0.25 mm. Ethylene glycol is used in the numerical simulation model with the following properties: density = 1109 kg/m^3^, viscosity = 20 mPa·s, surface tension = 0.048 N/m, dielectric constant = 37, electrical conductivity = 76 × 10^−6^ S/m. These parameters are kept the same in all simulations.

Due to geometry of the nozzle-substrate structure in Figure 2a, the distributions of the electric field and flow field in space are axisymmetric. The original three-dimensional model can be simplified as a two-dimensional axisymmetric model by reducing the calculation amount. According to the physical model, it is necessary to consider all influences of gravity, viscous force, surface tension, and electric field force. Considering these, Figure 2b shows the schematic diagram of a three-phase calculation model in the simulation. There are five boundary conditions that need to be specified. Boundary 1: inlet flow boundary, stagnation pressure, volume fraction of liquid = 1, uniform velocity $\vec{u_{z}}=u_{0}$, where $u_{0}=Q/A$ (A is cross-sectional area of nozzle and Q is flow rate). Boundary 2: no slip boundary, $\vec{u_{r}}=\vec{u_{z}}=0$ ($\vec{u_{r}}$ and $\vec{u_{z}}$ are the radial and axial velocity components, respectively), electric potential $\Phi =\Phi_{0}$ ($\Phi_{0}$ is applied potential). Boundary 3: continuative boundary, allowing liquid to flow out. Boundary 4 (ground electrode): no slip boundary $\vec{u_{r}}=\vec{u_{z}}=0$ electric potential Φ=0. Boundary 5: axisymmetric boundary.

After setting boundary conditions, high qualified mesh is also needed. In the numerical simulation model, the mesh adopts non-uniform mesh based on the "free mesh method" for achieving the high precision and small memory required for calculation. Due to the small size of the jet, a local grid of the central symmetry axis is encrypted with a minimum cell size ~0.5 μm for capturing accurate cone jets. In order to reduce the calculation amount, the initial shape of the liquid is added as a half sphere on the nozzle aperture. The time step should be no more than 5 ns to achieve a convergent solution after multiple attempts. The initial conditions are: t=0, for liquid $\vec{u_{z}}=u_{0}$, q=0, and applied potential on the nozzle is $\Phi =\Phi_{0}$. Calculation can be performed after all initial conditions are set correctly.

### 2.3. Results and Discussion

When the potential difference is 2.3 kV and the inlet flow rate is 200 nL/min, the whole process of droplet generation is successfully simulated in one whole cycle, including Taylor cone generation, jet onset, jet break, and jet retraction after several tens of hours of calculation. Volume fraction distribution, velocity distribution, and charge density distribution at different moments during E-jet printing can be also obtained.

**Figure 3:**
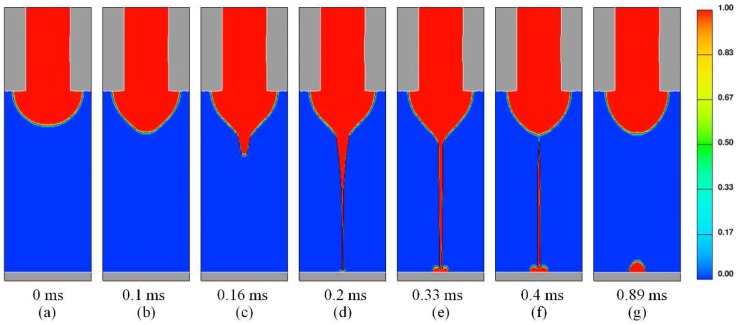
(**a**–**g**) Volume fraction distribution at seven representative moments during a whole injection cycle.

Figure 3a–g shows volume fraction distributions at seven representative moments during a whole injection cycle. The red region has a volume fraction of 1, which represents liquid, and blue regions have a volume fraction of 0, which represents air. The region between the two areas represents the gas/liquid interface. In simulation, an initial hemispherical meniscus (a) will continue to elongate with time increase, then a cone jet forms at the tip of the meniscus (b) and ejects (c). After the jet impinges on the substrate, some liquid will start to deposit. Then, the deposited droplet has a tendency to spread in the horizontal direction due to the inertia of the high-speed jet (e). After a period of deposition, the jet will break at the transition zone of the cone (f). Then, the fractured jet will continue to fly toward the substrate and the deposited droplet will keep on accumulating. Due to the effect of surface tension, the unfolded droplet will retract and shrink into a uniform spherical crown droplet (g). In addition, the total cycle during one whole injection process in the simulation is about 0.89 ms.

**Figure 4:**
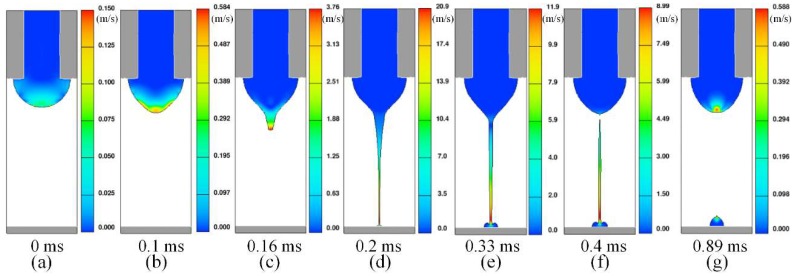
(**a**–**g**) Velocity distribution at seven representative moments during an injection cycle.

Figure 4a–g shows velocity distribution at the same seven representative moments during an injection cycle. The liquid in blue has very low velocity, while the red region has relative high velocity. When voltage is applied, meniscus starts to accumulate energy (a). The parts that have high velocity gradually move from the bottom to the top of meniscus (b). The velocity at the top of meniscus will become larger and larger, which drives formation of the Taylor cone. When the velocity reaches about several meters per second, a jet is ejected from the Taylor cone (c). With the effect of the electric field force, the jet will be accelerated continually (d). The highest velocity can even exceed 10 m/s. Thereafter, more liquid will eject from the cone (e). At this time, there is a huge speed difference between the jet and cone. At some point, the jet will break and separate from the meniscus (f). After a period of time, the point of the meniscus with maximum speed will reappear on the top and the next cycle of injection will start again (g). During the entire cycle, the point with maximum velocity always occurs at the point which is nearest to substrate. At first, the magnitude of the jet’s velocity increases gradually. However, it starts to decrease after the jet contacts the substrate. It can be concluded that the speed difference between the top part and the bottom part of the meniscus is a direct cause of the jet injection. In addition, the maximum velocity of the jet can reach an order of several m/s.

**Figure 5:**
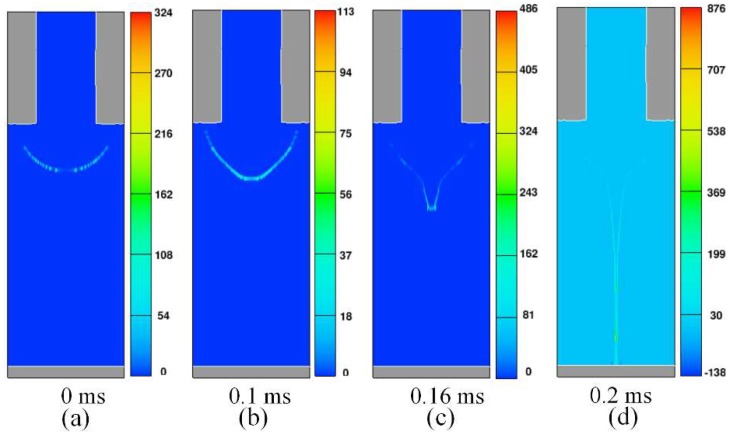
(**a**–**d**) Charge density distribution at four representative moments during an injection cycle.

Moreover, Figure 5a–d shows the change of charge density distribution at four representative moments during an injection cycle. From the figure, we know that charges always locate at the gas-liquid interface to form a surface charge layer (a). With time flows, charge density also gradually increases (b, c). When a jet which is carrying a large amount of charge contacts the substrate, charges at the tip of jet will rapidly transfer to the substrate (d), resulting in a decrease of charge density in the meniscus. Due to the charge relaxation phenomenon [34], the charges in the cone are greatly reduced and it is difficult to replenish them in a short time. Therefore, the solution in the meniscus cannot be replenished into the jet in time, and there will be a short supply imbalance. This is another perspective to explain the jet break. After the jet breaks, charges in meniscus will accumulate again. When the threshold of the charge density in the cone is reached, another jet will be dragged out.

## 3. Validation of Droplet Generation Process by Experiments

**Figure 6:**
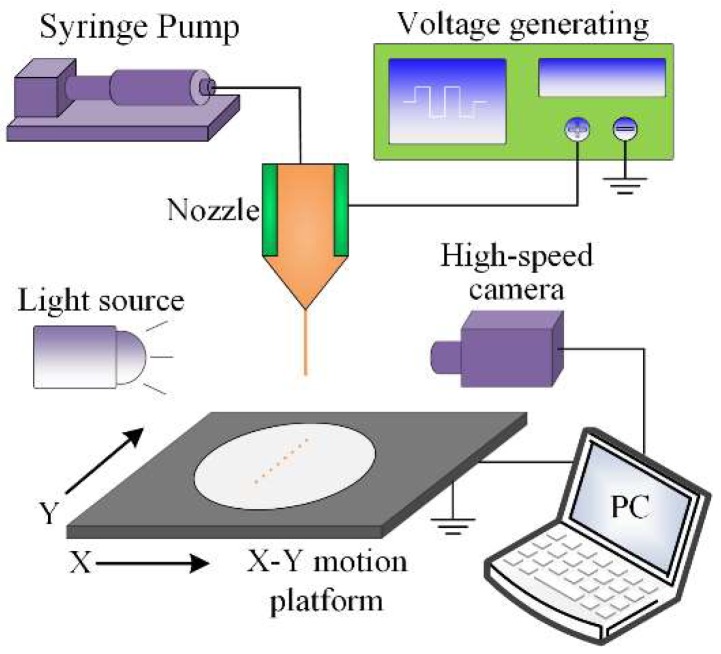
Schematic diagram of electrohydrodynamic jet (E-jet) printing system for experiment.

In this section, a stainless-steel nozzle was utilized to validate the feasibility of the proposed numerical simulation model by the E-jet printing experiment. Figure 6 shows a schematic diagram of the E-jet printing system for the experiment, including a 30G stainless nozzle, solution supply module, voltage-generating source, jet observation module, and motion platform. The solution was stored in a syringe that was connected and delivered to the inlet of the nozzle at a constant flow rate by a precision flow pump (11 Pico Plus, Harvard Apparatus, Inc., Holliston, MA, USA). The voltage-generating source consisted of a function generator (Agilent, 33500B, Keysight, Calabasas, CA, USA) and a high voltage amplifier (Trek, 609E-6, TREK, Inc., Lockport, NY, USA). The jet observation module included a high-speed camera (Cooke, Pco. dimax HD, PCO AG, Kelheim, Germany) with a microlens and a Halogen lamp (200 W) to capture the jetting process. For clearer vision, the experimental phenomenon was recorded at 10,000 frames/s. The Si substrate was placed on the surface of the X-Y motion platform to gather the fallen jet. The software, which operates and controls the X-Y motion platform, was written by VC++ language and Microsoft Foundation Class (MFC) Library.

In all experiments, the solution and process parameters were the same as the numerical simulation. The solution is ethylene glycol, with the following properties: density = 1109 kg/m^3^, viscosity = 20 mPa·s, surface tension = 0.048 N/m, dielectric constant = 37, electrical conductivity = 76 × 10^−6^ S/m. The substrate was single polished silicon wafer and the dielectric constant was 11.9. The distance from nozzle to substrate was 0.65 mm. The thickness of the substrate was 0.25 mm. The surface of the silicon substrate was modified to be hydrophobic by octadecyltrichlorosilane (OTS), with a contact angle of about 80°. The printed droplet or pattern on the hydrophobic substrate was more regular and finer and the volume of the droplet on the hydrophobic substrate was easily calculated. Room temperature and relative humidity are 25 °C and 30%, respectively. Printed droplets were observed by a confocal laser scanning microscopy (VK-X200, Keyence, Inc., Osaka, Japan).

**Figure 7:**
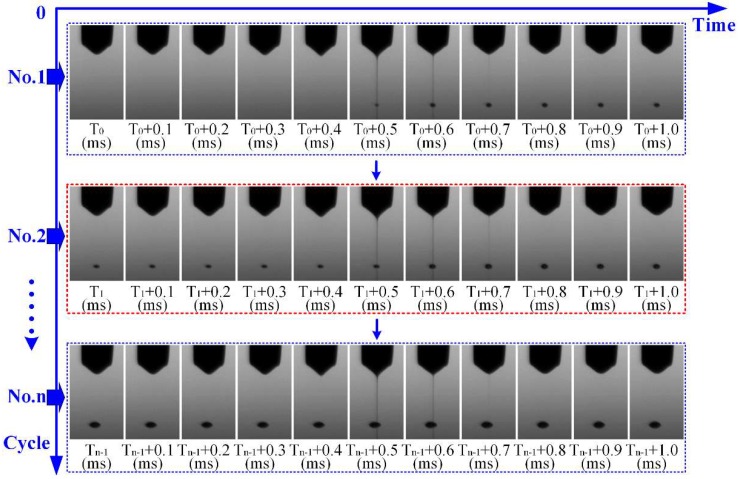
Image sequences of E-jet printing process in multiple cycles.

Figure 7 shows image sequences of the E-jet printing process in multiple cycles that was captured by high-speed camera in the experiment. The process parameters are: flow rate 200 nL/min, nozzle height 0.65 mm, applied high voltage 1.7 kV, and applied time for 50 ms in every second. In Figure 7, the horizontal axis represents time and the vertical axis represents the number of cycles. At the beginning (T_0_ ms), the meniscus hanging on the end of the nozzle starts to stretch gradually and form a Taylor cone (T_0_ + 0.4 ms) under the effect of the electric field. Then, a fine jet will occur when the electrical stress overcomes the surface tension (T_0_ + 0.5 ms), and flies to the substrate at a high velocity. After hitting the substrate, the jet will be thinner and thinner (T_0_ + 0.6 ms). At a suitable moment, the jet will break near the tip of the cone (T_0_ + 0.7 ms), rush to the substrate (T_0_ + 0.8 ms), and mix together with the previously accumulated droplet. After a final droplet occurs on the substrate, the meniscus will retract to the initial position (T_0_ + 1 ms) and the whole E-jet printing process of one cycle is completed. During the applied period of high voltage, the four processes repeat continuously, including Taylor cone generation, jet injection, jet break, and meniscus retraction. As shown in Figure 7, after a period of time (the charge re-accumulates in the solution), the second cycle of the E-jet injection process starts at the time *t* =T_1_ ms. A new jet will form and accumulate with the original droplet. In the second cycle, all the details are almost the same with as the first cycle until *t* = T_1_ + 1 ms. Finally, a cumulative droplet forms on the substrate after the N_th_ injection cycle is completed. By regulating the duration and frequency of the applied voltage, a controllable droplet array can be achieved by drop-on-demand E-jet printing.

Comparing with numerical simulation results, we can conclude that simulations and experiments are in good agreement with each other in many aspects, such as period, velocity magnitude, four steps in an injection cycle, and shape of jet in each step. Therefore, the established numerical simulation model is effective and accurate, and can be utilized to investigate influences of structural parameters and control parameters of nozzle controlling parameters for highly efficient and precise E-jet printing.

## 4. Influences of Controlling Parameters in E-Jet Printing

The proposed numerical simulation model can be utilized, not only to analyze internal mechanisms of jet generation, jet fracture, and jet re-formation, but also to investigate influences of important controlling parameters. The relationships behind this help to achieve design constraints and guide printhead design. In this section, further simulations are performed to reveal influences of applied voltage, flow rate, and nozzle diameter.

**Figure 8:**
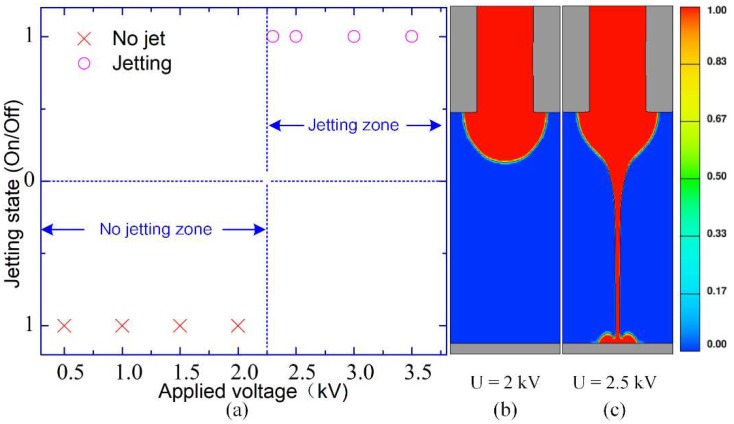
(**a**) Relationship between jetting state and applied voltage, (**b**,**c**) Images of meniscus shape at the same calculating moment (*t* = 0.4 ms) in 2.0 kV and 2.5 kV, respectively.

At first, to analyze influences of applied voltage, a 30G nozzle is utilized and eight different voltages are applied (0.5 kV, 1.0 kV, 1.5 kV, 2.0 kV, 2.3 kV, 2.5 kV, 3.0 kV, and 3.5 kV). The working height is 0.65 mm while the liquid flow rate is 200 nL/min. Jetting states in eight applied voltages are recorded by comparing images of meniscus shape at the same calculating moment (*t* = 0.4 ms), as shown in Figure 8a. The total calculating time in each simulation is 3 ms, and two representative states under 2.0 kV and 2.5 kV are demonstrated in Figure 8b,c, respectively. When the voltage is 2.0 kV or less than 2.0 kV, no jetting occurs at this moment (*t* = 0.4 ms), even though the meniscus begins to grow. When the voltage is 2.3 kV or larger than 2.3 kV, jetting can take place. It indicates that the magnitude of the applied voltage determines whether jetting can start or not.

Secondly, in order to analyze influence of flow rate on E-jet printing, a fixed voltage of 2.3 kV and seven different flow rates (0 nL/min, 500 nL/min, 1000 nL/min, 1500 nL/min, 2000 nL/min, 2500 nL/min, and 3000 nL/min) are applied. The total calculating time in each simulation is 3 ms. The working height is 0.65 mm When the flow rate is 0 nL/min, only one instance of jetting will occur and no jetting will follow the first one, even after a long calculating time. When the applied flow rates are 500 nL/min or larger than 500 nL/min, multiple jets will appear continuously from the meniscus. It proves that flow rate decides whether the injection process can take place steadily and continuously or not.

**Figure 9:**
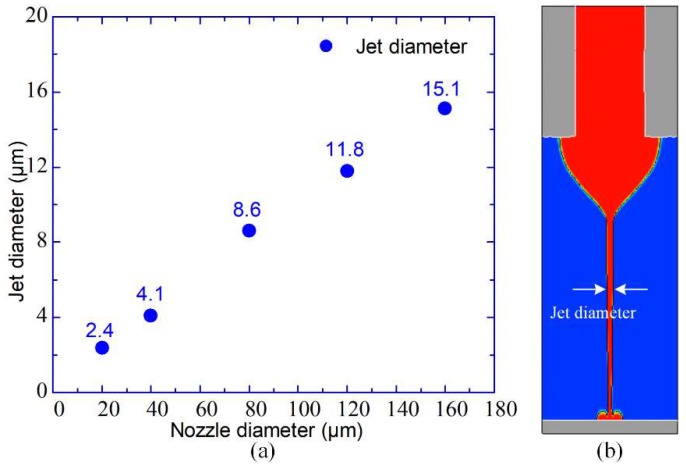
(**a**) Relation between nozzle diameter and jet diameter. (**b**) An example of a jetting image that is adopted to measure jet diameter when a 160 μm nozzle is utilized.

Thirdly, jet diameter can directly affect the precision and accuracy of the printed structure when E-jet printing is utilized for preparation of micro scale functional structures. However, jet diameter is not a parameter that can be modulated directly like nozzle diameter. Therefore, it is significant to analyze how nozzle diameter affects jet diameter for perusing high printing resolution. Here, a fixed nozzle to substrate distance of 0.65 mm, a fixed flow rate of 1000 nL/min, and five different sized nozzles (20 μm, 40 μm, 80 μm, 120 μm, and 160 μm) are utilized. Actually, the applied voltages are the onset voltages of each situation. When the nozzle diameters are 160 μm, 120 μm, 80 μm, 40 μm, and 20 μm, the applied voltages are 2.3 kV, 2.1 kV, 1.9 kV, 1.6 kV, and 1.4 kV, respectively. Jet diameters are measured in each situation and the relationship between nozzle diameter and jet diameter is expressed in Figure 9a. The jets of a few micrometers are measured by two steps. At first, the jetting image in every sequence of the jetting process is recorded. Then, a simple image processing method is utilized against the photo in which the jet is connecting the meniscus and substrate to measure the jet diameter. Figure 9b shows an example of a jetting image that is adopted to measure jet diameter when a 160 μm nozzle is utilized. We can conclude that the smaller the nozzle diameter, the finer the jet. In another view, the nozzle diameter determines the ultimate resolution of E-jet printing. To achieve higher printing resolution, nozzle diameter needs to be designed as small as possible.

In a word, it is necessary to consider the following three design constraints when designing an E-jet printing printhead: (1) voltage constraint—applied voltage should be larger than the critical onset voltage; (2) flow rate constraint—the proper amount of solution should be continuously and stably supplied to the nozzle. (3) nozzle diameter constraint—if possible, a nozzle with a small diameter is demanded for achieving ultimate resolution of the micro functional structure. Therefore, the established numerical simulation model provides a route to give theoretical guidance for optimal design of the E-jet printhead.

## 5. Conclusions

A numerical simulation model for the droplet generation process in E-jet printing is established based on the Taylor-Melcher leaky-dielectric model. The whole process of droplet generation is successfully simulated in one whole cycle, including Taylor cone generation, jet onset, jet break, and jet retraction. Compared with experiments, period, velocity magnitude, four steps in an injection cycle, and shape of jet in each step are in good agreement. This confirms the validity and accuracy of the proposed numerical simulation model. Further analysis reveals three constraints that need to be considered: (1) voltage constraint—applied voltage should be larger than the critical onset voltage; (2) flow rate constraint—the proper amount of solution should be continuously and stably supplied to the nozzle; (3) nozzle diameter constraint—if possible, a small sized nozzle is needed for achieving ultimate resolution of the micro functional structure. The established numerical simulation model provides a useful tool to deeply recognize E-jet printing and guide the design of printheads for E-jet printing systems with high performance in the future.
